# Men's Economic Abuse Toward Women in Sweden: Findings From a National Survey

**DOI:** 10.1177/10778012241257248

**Published:** 2024-06-06

**Authors:** Linnéa Bruno, Sofia Strid, Hans Ekbrand

**Affiliations:** 1 Department of Child and Youth Studies, 7675 Stockholm University, Stockholm, Sweden; 2 Department of Sociology and Work Science, 3570 Gothenburg University, Gothenburg, Sweden

**Keywords:** economic abuse, financial abuse, intimate partner violence, prevalence, Sweden

## Abstract

Drawing from a nationally representative survey (*n* = 6,611), this article analyses the prevalence of men's economic abuse toward women in Sweden. Economic abuse is still a relatively marginalized area of research but is increasingly recognized as a distinct type of intimate partner violence. A few Swedish studies have specifically focused on economic abuse, yet none of which with a quantitative approach. A main finding is that motherhood significantly increases the risk of exposure. Furthermore, women report economic abuse from expartners (25%) to a much greater extent than from current partners (8%).

## Introduction

Men's violence against women in intimate relationships is a multifaceted and pervasive problem, globally affecting one-third of women (WHO, 2021). In Sweden and the Nordic countries, as elsewhere, men's violence against women is defined and conceptualized as a cause and consequence of gender inequality. Since 2006, ending men's violence against women has been included in national gender equality strategies and measures. The current governmental 10-year national strategy on preventing men's violence against women (Skr. 2016/17:10) evaluates previous work and serves as a guidance framework. Despite decades of equality-promoting policies and reforms to counteract this violence, women continuously report high levels of violence. In the largest European prevalence survey on men's violence against women (*n* = 42,000; FRA, 2014a), nearly half of the Swedish respondents reported having been subjected to sexual and/or physical violence since the age of 15. More recently, a Swedish national prevalence study (*n* = 6,500) confirmed similar levels: 55% of the women reported being subjected to physical, sexual, psychological, or economic since the age of 15 (Strid et al., 2023). These rates echo the results of the first Swedish prevalence study, conducted more than 2 decades years ago (Lundgren et al., 2001).

Intimate partner violence (IPV) is one of the most common forms of men's violence against women (FRA, 2014a; WHO, 2021). For some IPV survivors, the violence and abuse end with separation or divorce. For others, the abuse continues, escalates, or takes new forms such as stalking, threats and economic abuse during and following separation (c.f. Humphreys et al., 2019; Katz et al., 2020; Spearman et al., 2022). Not least may this be the case when the perpetrator and the survivor share responsibility for dependant children. Policies and professional discourses and practices within family law, social services and social insurance may aggravate inequalities and increase abusers’ scope for action, several studies suggest (c.f. Cook et al., 2015; Natalier, 2018; Tegler et al., 2023). Even in a welfare state such as Sweden, high demands are placed on divorced/separated survivors to take personal responsibility when financially vulnerable because of IPV (Ulmestig & Panican, 2016). Survivors with children are often required to cooperate with the perpetrator/father after separation, which implies a risk of further abuse. Out of fear of continued violence, or of concern for negative effects on father–child relationships, victimized mothers may lower their demands in negotiations, avoiding arguing over money (Bruno, 2018a; Ekbrand, 2006). Notwithstanding, qualitative interviews with mothers also include examples of valuable support from social services, emotionally as well as financially (Ulmestig & Eriksson, 2016). As recently noted by the Swedish Gender Equality Agency (2023) there seems to be an increased awareness among authorities and practitioners, working in this field, of economic abuse as a widespread type of violence with serious consequences. Nordic research focusing on this particular type of IPV, however, is still scarce. Economic abuse may have long-term effects on those exposed to it such as poverty, ill health, precarious or unsafe housing, child neglect, and delinquency (Branigan, 2004; Eriksson & Ulmestig, 2021; Huang et al., 2015). On a societal level, the consequence when this type of IPV goes unnoticed by authorities is that intersectional inequalities may grow deeper.

Our point of departure is feminist violence theory, which differs from the conventional one grounded in criminological or psychological violence research in at least three aspects: the forms of violence included, its explanatory model, and the analysis of the direction of violence (Strid & Meier-Arendt, 2020). In contrast to conventional psychology and criminology, which often focus on direct, individual, intentional, and often physical harm, feminist violence theory analyses violence as a physical, psychological, emotional, sexual, economic, and social phenomenon and focuses on effects, rather than intentions (Strid et al., 2021). In the conventional model, the violence is explained through the perpetrator's position as relatively marginalized, exposed, or socially maladjusted, while the feminist analysis makes visible the relative power and privileges of the perpetrator. According to the former, violence is perpetuated by the relatively powerless against those with relative power, while the direction of violence is reversed in the feminist theory of violence. The privileges of the perpetrator's position, for example, regarding finances, work, ethnicity, language, and social networks, compared to the victim’s position are thus highlighted (Bruno, 2018b; Strid et al., 2013; Walby et al., 2017). In essence, the feminist continuum-thinking, and broad understanding of violence grounded in survivors’ experiences differ from an incident model in that it acknowledges how various types of abuse tend to have mutually reinforcing and cumulative effects (Kelly, 1988). For example, financial control may be used to isolate and further psychologically and sexually abuse and exploit the victim. Out of fear of physical violence or because of visible marks from such violence, the victim may avoid going to class or work outside the home, which in turn makes her more isolated and disempowered, financially as well as psychologically.

This article aims to analyze the prevalence and characteristics of men's intimate partner economic abuse against women in Sweden. It addresses the following research questions: (a) What is the overall prevalence and which type of economic abuse is most prevalent? (b) What are the relations/overlaps between economic abuse and physical and sexual violence? (c) Are there significant differences in victimization between separated and not separated women? (d) Are there significant differences between women with and without children?

## Previous Research on Economic Abuse

Economic abuse is defined by the United Nations (2006), the Council of Europe (2011), the European Commission (2022) and scholarship (c.f. Adams et al., 2008; Postmus et al., 2016; Stylianou, 2018) as a form of men's violence against women. It can include control, exploitation, sabotage, or neglect that harms the victim financially, and may include both criminal and legal acts. Importantly, the violence can be more or less intentional (Stark, 2007). An often-cited, but more narrow definition of this type of IPV is “behaviours that control a woman's ability to acquire, use and maintain economic resources, thus threatening her economic security and potential for self-sufficiency” (Adams et al., 2008, p. 564). Similarly, Kutin et al. (2017, p. 269) define economic abuse as “behaviours aimed at manipulating a person's access to finances, assets and decision-making to foster dependence and control.”

### Prevalence Studies

Most large-scale studies on IPV, or violence more broadly, exclude items explicitly measuring economic abuse. Some only include one or two items measuring this type of IPV. Two decades ago, the first large-scale study on men's violence against women in Sweden was conducted (Lundgren et al., 2001). In this study, being denied influence over the family's economy was categorized as a form of psychological violence: 10% of the 7,000 surveyed women reported having been subjected to this type of abuse by a current or former partner (Lundgren et al., 2001). In the present article, we draw from a national survey which measures economic abuse using five items (detailed in the method section). To date, there is no agreed-upon index with which to measure economic abuse. The first attempt at a full scale for this type of IPV, the Scale of Economic Abuse (SEA-28; Adams et al., 2008) was later developed by Postmus et al. (2016) into a scale of 12 items covering three distinct areas: *financial control, economic exploitation,* and *employment sabotage* (SEA-12). A meta-analysis found that financial control was the most researched form (Postmus et al., 2020). *Financial control* can be exemplified by denying access to money/accounts, refusing to contribute financially to purchases, keeping secret financial information, and controlling/determining household expenses. The second most common form was *economic exploitation*, which can take the form of squandering the household's money; theft of property, money, or identities; coerced debt; foreclosing/firing out the partner from the home, sale of the partner's property or things that are necessary for the household, refuse the partner's care, insurance, or transport. The third form, *employment sabotage*, is about preventing the partner from going to work or interfering with the partner at work. The SEA-12 has been validated in English and recently also in Spanish (Johnson et al., 2021). Economic abuse often seems to overlap with other forms of IPV (Gottlieb & Mahabir, 2021; Haeseler, 2013; Stylianou, 2018). Among service-seeking samples from the United States and the United Kingdom, 76%–99% of survivors report having experienced economic abuse (Adams et al., 2015; Postmus et al., 2012, 2016; Stylianou et al., 2013). In a South African study, 45% of women reported having been subjected to a combination of emotional and economic abuse (Gibbs et al., 2018). In an Indian survey with more than 4,900 women aged 18–49 years, 23% reported experiences of at least one form of economic abuse (Kanougiya et al., 2021). The lifetime prevalence of economic abuse in an Australian population sample (*n* = 17,000) was 12%. Women in all age groups were more likely to experience economic abuse (16%) compared to men (7%; Kutin et al., 2017). A previous Australian study suggested that 89% of women who had been involved in postseparation family law disputes reported having been subjected to financial abuse (Bagshaw & Brown, 2010). A study with 1,823 women who called the National Domestic Violence Hotline in the United States revealed that more than half, 52%, reported coerced debt (Adams et al., 2020). Analyzed data from 8,478 women in the Philippines suggested employment sabotage to be the most prevalent type of economic abuse, “ever lost job/source of income because of husband” (7%), “not allowed to engage in legitimate work” (4%; Antai et al., 2014).

### Economy and IPV in Different Contexts

Like other types of IPV, economic abuse has different implications for different survivors (Bruno, 2022; Leigh Doyle, 2020; Serpa Pimentel et al., 2021). As Sanders (2015, p. 23) concludes, women are subjected to economic abuse “not only when their resources are low and their dependence high.” Relatively few studies on violence place economic violence at the center of the analysis. In research on violence and economy, it is more often the lack of resources as an obstacle to leaving a violent partner, or the effects of violence on the victim's finances, which are investigated. Regarding the relationship between women's economic independance and exposure to violence, international research has come to conflicting results (Hughes et al., 2015). On the one hand, own/increased income can strengthen the woman's position toward a male partner, improve relationships and reduce the risk of violence (Haile et al., 2012). A metaanalysis of nearly 50 quantitative and qualitative studies on the separation processes of abused women showed that the lack of financial resources was the most common obstacle to leaving a violent partner (Andersen & Saunders, 2003). On the other hand, women's increased financial independance in a relationship may also increase the risk of physical and sexual violence, and men can use violence to prevent women from paid work (Sanders, 2015; Voth Schrag, 2015). Recent studies from China (Li et al., 2023) and from the Dominican Republic (Caridad Bueno & Henderson, 2017) show a significantly higher risk for a woman to be subjected to sexual violence from a male partner when her salary is higher than his. Few Swedish studies have examined economic abuse, none of which hitherto with a quantitative approach to examine its prevalence (Bruno, 2018a; Eriksson & Ulmestig, 2021). However, recent research within the field of critical welfare studies suggests that reforms in Swedish family policy—aiming to promote parental cooperation postseparation—instead may contribute to the reactualization of IPV and enhance financial control (Fernqvist et al., 2023; Fernqvist & Sépulchre 2022; Tegler et al., 2023).

### Separation and Economic Abuse

As revealed by a growing number of studies from diverse contexts, economic abuse seldom ceases to exist postseparation, even if other types of violence may have ended (Branigan, 2004; Fawole, 2008; Krigel & Benjamin, 2021; Spearman et al., 2022). Similar to other types of IPV, separated women report higher prevalence. Up to 90% of those who have separated from a violent partner in the United States report continued harassment, stalking, or abuse (Hardesty et al., 2012; Mitchell et al., 2021). In Finland, a Nordic welfare state in many ways similar to Sweden, qualitative interviews with women include examples of four types of postseparation economic abuse: economic sabotage (destroying possessions and sabotaging employment), withholding resources (e.g., prolonging the divorce process, refusing to divide assets or to pay child support), financial harassment (e.g., false accusations, threatening social networks) and stealing (Kaittila et al., 2024). Some forms of economic abuse obviously arise following separation, such as withholding child support, manipulating authorities, using family law and other legal proceedings to increase financial control (Bruno, 2018a; Fernqvist & Sépulchre, 2022; Fernqvist et al., 2023; Galántai et al., 2019; Lynch et al., 2021; Miller & Smolter, 2011; Toews & Bermea, 2017). However, having separated may also enhance a clearer understanding of the previous relationship, viewing the perhaps then normalized abuse from a more critical perspective. In addition, breathing space and a sense of safety may increase courage and thus the capacity needed for disclosure of violence (Brännvall, 2016; Ekbrand, 2006; Enander, 2008). In sum, the overrepresentation of experiences of violence among separated women may presumably in part be explained by increased ability to disclose/report after having left an abusive partner. To which extent there is also an actual increase in violence postseparation is however unclear.

### Consequences of Economic Abuse

Economic abuse is still not always recognized as a specific type of violence, but indeed often categorized as an expression of control or psychological violence. Categorizing economic abuse solely as a form of psychological violence or coercive control, however, may obscure the severe consequences of this abuse, on physical health and material conditions. It has been found to imply a greater risk of depression and suicidal thoughts than exposure to physical or sexual violence. Multiexposure, however, entails the greatest risk (Gibbs et al., 2018). Drawing from longitudinal data in the United States, Gottlieb and Mahabir (2021) found evidence that mothers who have been subjected to IPV are at heightened risk of being involved in criminal justice. Economic and physical abuse had a stronger independant association with involvement in criminal justice, in comparison to sexual and emotional abuse.

The spatial component is a central dynamic, since abusers may exert economically abusive tactics without any contact or physical proximity with the victim (Stylianou, 2018). Furthermore, economic abuse may have severe and longstanding consequences, such as depression, child maltreatment, economic hardship, and poor physical health (Johnson et al., 2022; Postmus et al., 2012). Especially as regards economic abuse, there is thus a continuum over time and space (Eriksson & Ulmestig, 2021). Not least economic abuse negatively affects already vulnerable children. A particularity with research on this type of IPV is that children's perspectives are missing in the vast majority of studies on economic violence, in contrast to the relatively extensive body of research on children's experiences of having been exposed to physical IPV (Bruno, 2022; Näsman & Fernqvist, 2015).

## Methods and Material

The article is based on quantitative data from a nationally representative prevalence survey on men's violence and women's safety in Sweden (Strid et al., 2023). The survey included questions about women's experiences of different forms of men's violence, including control, threats, physical, psychological, sexual violence, and abuse, in online/offline contexts, in different relationships in the most recent year and since the age of 15. Regarding economic abuse, however, the questions refer to any time since the age of 15, and from current and former partners respectively.

### Data Collection Method

The survey was administered by Statistics Sweden from April to August 2021. It was sent to an unattached random sample of 14,967 women aged 18–86, and 6,611 women completed the survey, giving a response rate of 44%. The response rate is higher from older women aged 75–84 (52%) lower from younger women aged 18–24 (34%), highest in the 64–74 group (60%), and higher for women born in the Nordic countries and Europe, than for women born outside of Europe. The survey responses were supplemented by SCB with register data for information on age, country of birth, highest level of education achieved, net income and total earned income, main source of income, municipality, and age of any children.

Generally, a response rate of approximately 50% is considered normal for surveys. However, a high response rate per se does not guarantee high quality, nor does a relatively low response rate necessarily imply low quality (Holtom et al., 2022). Furthermore, it is common to anticipate lower response rates in research that involves sensitive subjects such as IPV (FRA, 2014b). Underreporting of abuse is often assumed, for example, because of recall bias of less severe forms of violence. In addition, severely victimized women still living with the perpetrator are particularly less likely to participate in research (Simmons & Swahnberg, 2019). The overall response rate of 44% is comparable to the response rates achieved in, for example, the EU-wide survey on violence against women (response rate 42.1%) conducted by the European Union Agency for Fundamental Rights, the European Quality of Life Survey (response rate 41.3%), conducted by the European Foundation for the Improvement of Living and Working Conditions (Eurofound, an EU agency) in 27 EU Member States, or the European Working Conditions Survey (response rate 44.2%), conducted by Eurofound in 27 EU Member States (FRA, 2014b). As is common with surveys, the sample may over- or underrepresent specific categories of respondents when compared to the whole population. Such variations are often handled through weighting. In the next section, the procedures of weighting used in the present study are detailed.

The survey included five items on economic abuse. Three of the five items were also used in the first Swedish national prevalence study (Lundgren et al., 2001) and two have been added based on the Scale of Economic Abuse 12 (Postmus et al., 2016). Combined, the five items capture economic exploitation, economic control, and employment sabotage, addressing economic abuse from a current male spouse/cohabiting partner or former male spouse/cohabiting partner. The questions were asked as descriptions of the woman's relationship to the man (current and former spouse/cohabiting partner), and the respondents had to agree or disagree with the descriptions with a binary response: yes or no.

*Economic exploitation:* (a) He forces you to borrow money, sign contracts or has used you in other ways to make money, and (b) He uses your or shared money to buy things for himself. *Economic control:* (c) You are not allowed to make decisions about money or buy things you want, and (d) He intentionally damages your belongings, a pet or other things you like. *Employment sabotage:* (e) He prevents you from working or studying outside of the home.

While these questions are concrete and relatively simple and based on previous research (as outlined above), violence is a phenomenon with vague boundaries. There is no perfect operationalization of violence. Regardless of which concrete questions are used to capture the concept of violence, interpretive work is required on the part of the respondent to produce the best possible answers to the questionnaire.

### Data Analysis Method

**Figure 1.**
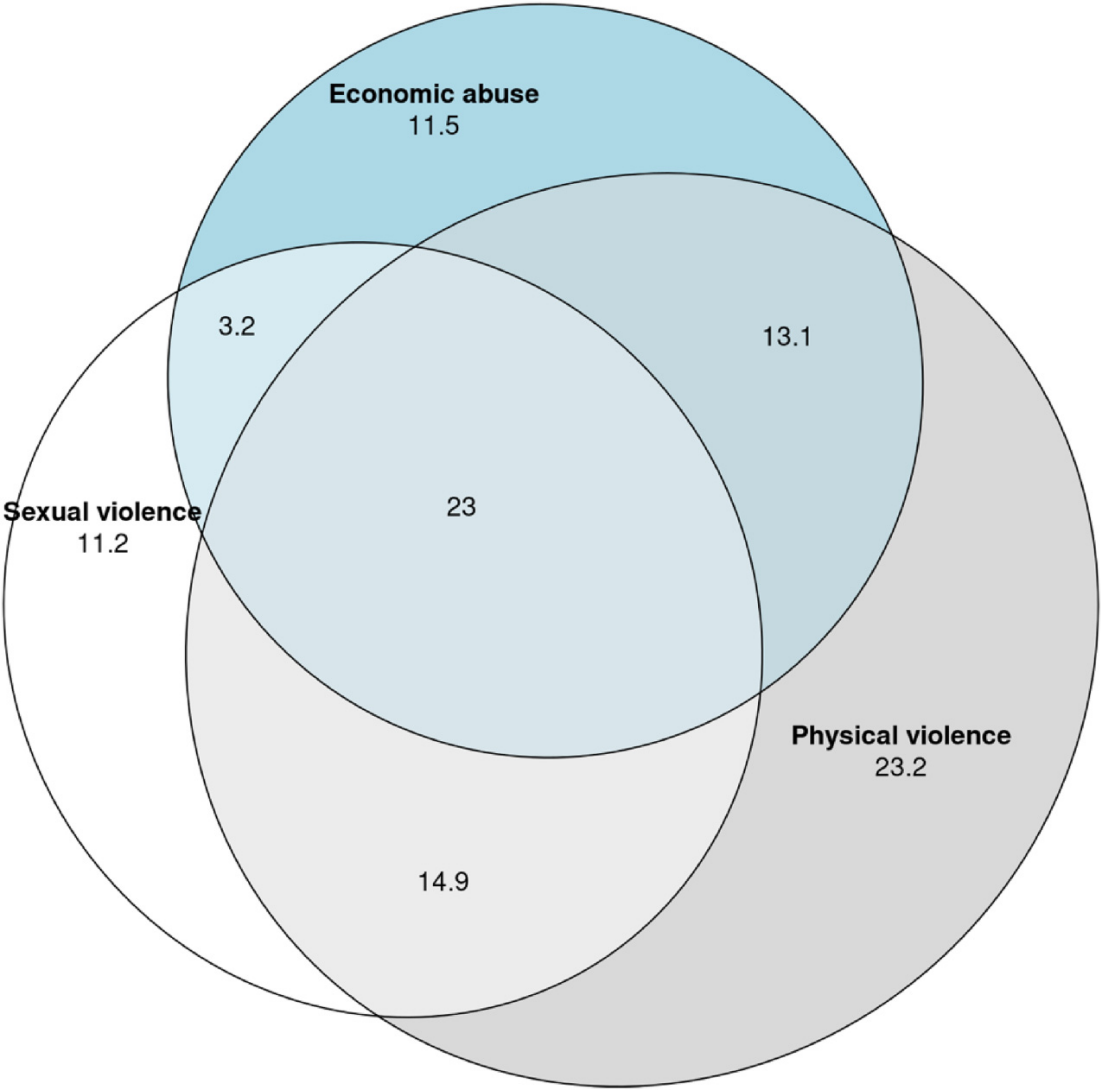
Euler diagram for the overlap between Sexual violence, Physical Violence and Economic abuse from a former spouse/partner. N = 3235, number of women exposed to at least one of these types of abuse = 1202. The numbers in the graph denote the percentage of the exposed who were exposed to the combination of forms of violence represented by the surface.

All data processing and analysis were conducted using the programming language R, and the corresponding code is available (Ekbrand, 2023; R Core Team, 2022). As stated above, the response rate exhibited noticeable variations based on age, country of birth, and marital status. Specifically, younger women, women born outside the Nordic countries, and unmarried women demonstrated a significantly lower response rate compared to older women, women born in the Nordic countries, and married women, respectively. To ensure that the descriptive analysis accurately represents the entire population, we applied weights generated by Statistics Sweden, which took into account factors such as age, country of birth, and marital status, during the univariate analysis (Lumley, 2020). However, we did not utilize weights in the regression analysis or in the examination of the overlap between different types of violence (Figure 1), as these estimates are not affected by any imbalances in response rates across various demographic groups, which could potentially result in a nonrepresentative proportion of women with experience of violence among those who responded. Furthermore, we generated numerous aggregated measures by combining responses from different questions. Typically, we created dichotomous aggregated measures, which indicate whether any of the underlying variables were satisfied or if all variable values indicated the absence of the measured phenomenon. Dichotomous aggregated measures offer the advantage of providing a percentage for all women (or for a specific subgroup of women) as victims. However, they also possess the disadvantage of obscuring differences in the level of vulnerability. Most of the findings are based on descriptive univariate and bivariate statistics (Venables & Ripley, 2002), but we also use inferential statistics in the form of logistic regression to estimate how economic violence varies as a function of motherhood, and the relation between the mother and the father(s) of her children. In these models, the age group of the woman is included to control for differences in the outcome related to age. The general form of those models is: $y=\beta_{0}+\beta_{1}motherhood+\beta_{2}\;agegroup_{1}+\beta_{3}\;agegroup_{2}+\ldots +\beta_{7}\;agegroup_{6}$ , where *y* is the logit of the probability of the woman being exposed to economic abuse, $\beta_{0}$ is the intercept, and $\beta_{1}motherhood$ is a dummy variable for the different states available which differ depending on if the abuser is a current or a previous spouse/partner. When the probability of the current partner to use economic abuse is modeled, motherhood has four possible states, and is thus covered by three dummy variables. $\beta_{k}age$ represents a set of dummy variables for seven age groups. When we evaluate the effect of motherhood we predict the probability of the outcome with $agegroup_{k}$ set to their mean values.

## Results

This section presents the results of the analysis. It first describes the overall prevalence and forms of economic abuse, in total and divided by former and current spouse/cohabiting partner. Then follows the overlaps between economic abuse and physical and sexual violence. Finally, the section analyses motherhood as a risk factor for economic abuse.

### Prevalence and Forms of Economic Abuse in Current and Previous Relationships

**Figure 2.**
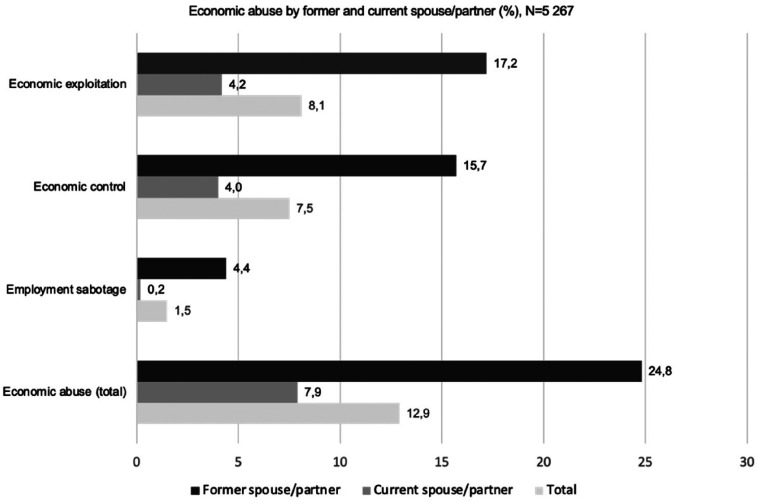
Economic abuse, by former and current spouse/partner and total.

**Figure 3.**
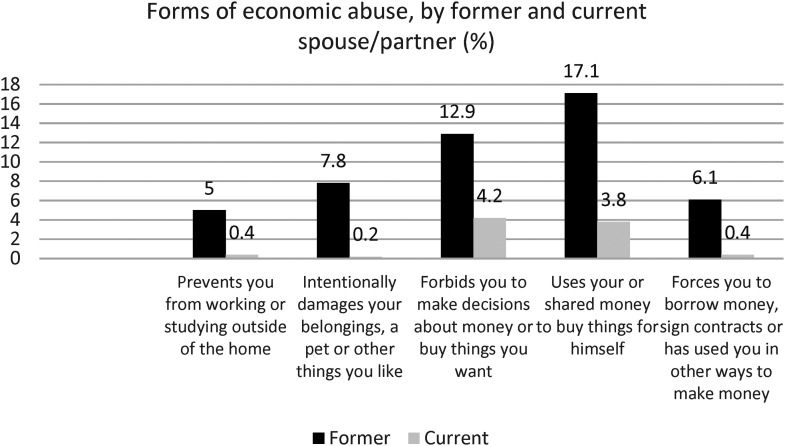
Forms of economic abuse, five items, by former and current spouse/partner and total.

The results show that 13% of women have been subjected to economic abuse. There are however major differences between women's experiences of economic abuse by a former or current spouse/partner: 8% of the respondents had experienced economic abuse by a current partner compared to 25% by a former partner (Figure 2). This difference holds for all forms of economic abuse: women experience higher levels of economic abuse by former partners than by current partners (Figures 2 and 3). Women are most likely to experience economic abuse as economic exploitation, followed by economic control, followed by employment sabotage. These results differ from previous research (Postmus et al., 2020), where the most common form of economic abuse is economic control.

Looking at the specific items within each of the three forms of economic abuse, the most commonly experienced form of economic abuse differs between former and current/partners. For the former partner, it is that he uses the woman's money or shared money to buy things for himself (17%), whereas for the current it is that he does not allow her to make decisions about money or to buy things she wants (4%; Figure 3).

### Overlaps Between Economic Abuse and Other Forms of Violence

Experiences of economic abuse overlap with experiences of other types of violence, including sexual violence and physical violence (see Figure 1). The combination of economic abuse and physical violence is more common than the combination of economic abuse and sexual violence. It is rather uncommon to have experienced only economic abuse, 11.5% report this, while the remaining 88.5% have experienced at least one additional form of violence, and 23% have experienced all three forms of violence.

### Motherhood and Fatherhood and Economic Abuse

**Figure 4.**
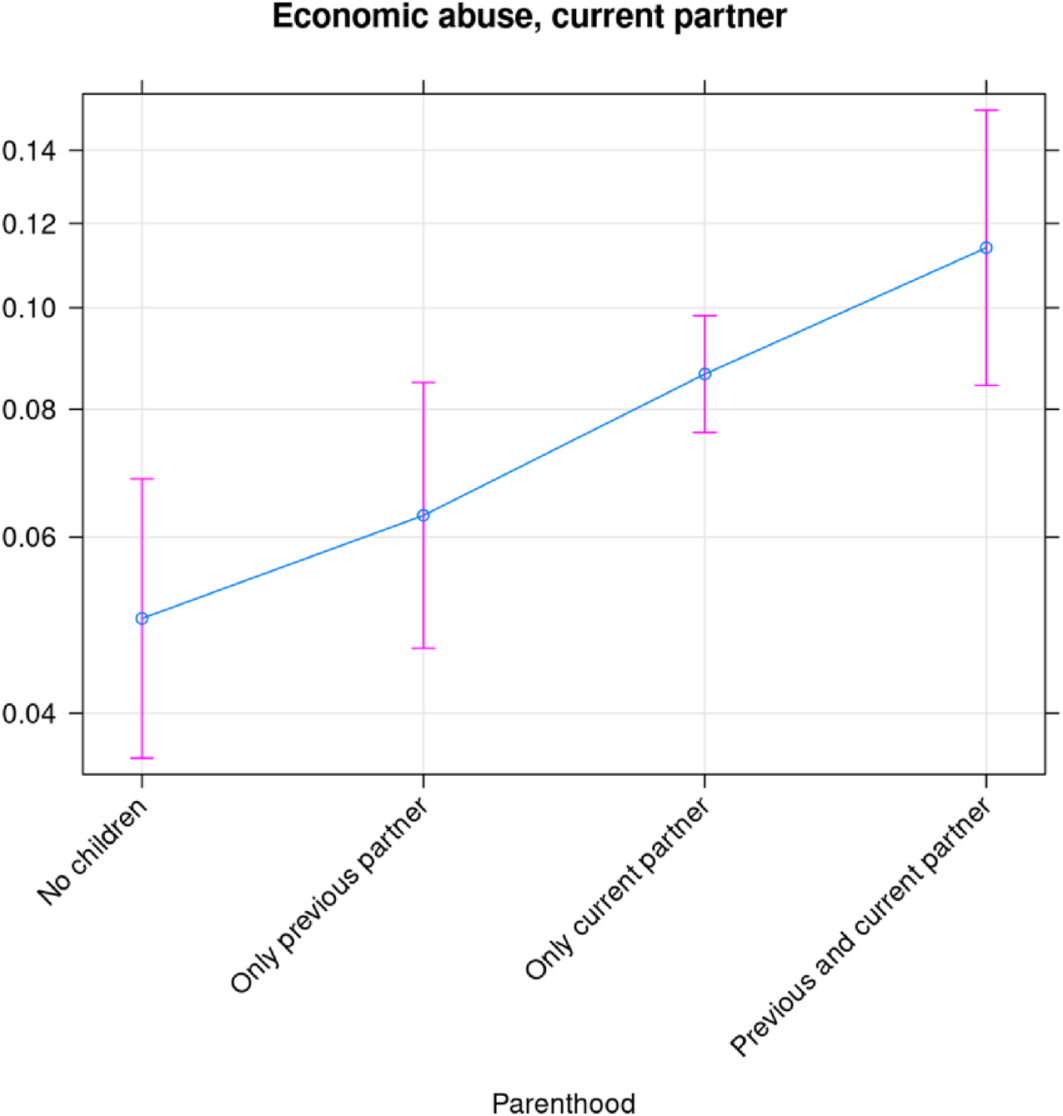
Probability of being exposed to economic abuse by the current spouse/partner as a function of parenthood.

Having children tends to increase the risk of economic abuse. There is a higher risk of economic abuse by the current partner if the woman has a child together with the current partner, compared to if she has no child (compare “no children” and “only current partner” [Figure 4]). All models in this section, except where it is noted otherwise, include a control variable that records the age group of the woman, and the predictions are produced when the control variable is kept at its mean value. The statistically significant differences in the risk of economic abuse from the current partner are between on the one hand “no children” and on the other hand “only current partner” and “former and current partner.” The difference between “only current partner” and “former and current partner” is not statistically significant, but the results suggest that having a child with a former partner increases the risk that the current partner uses economic abuse regardless of whether the woman has children together with the current partner or not.

**Figure 5.**
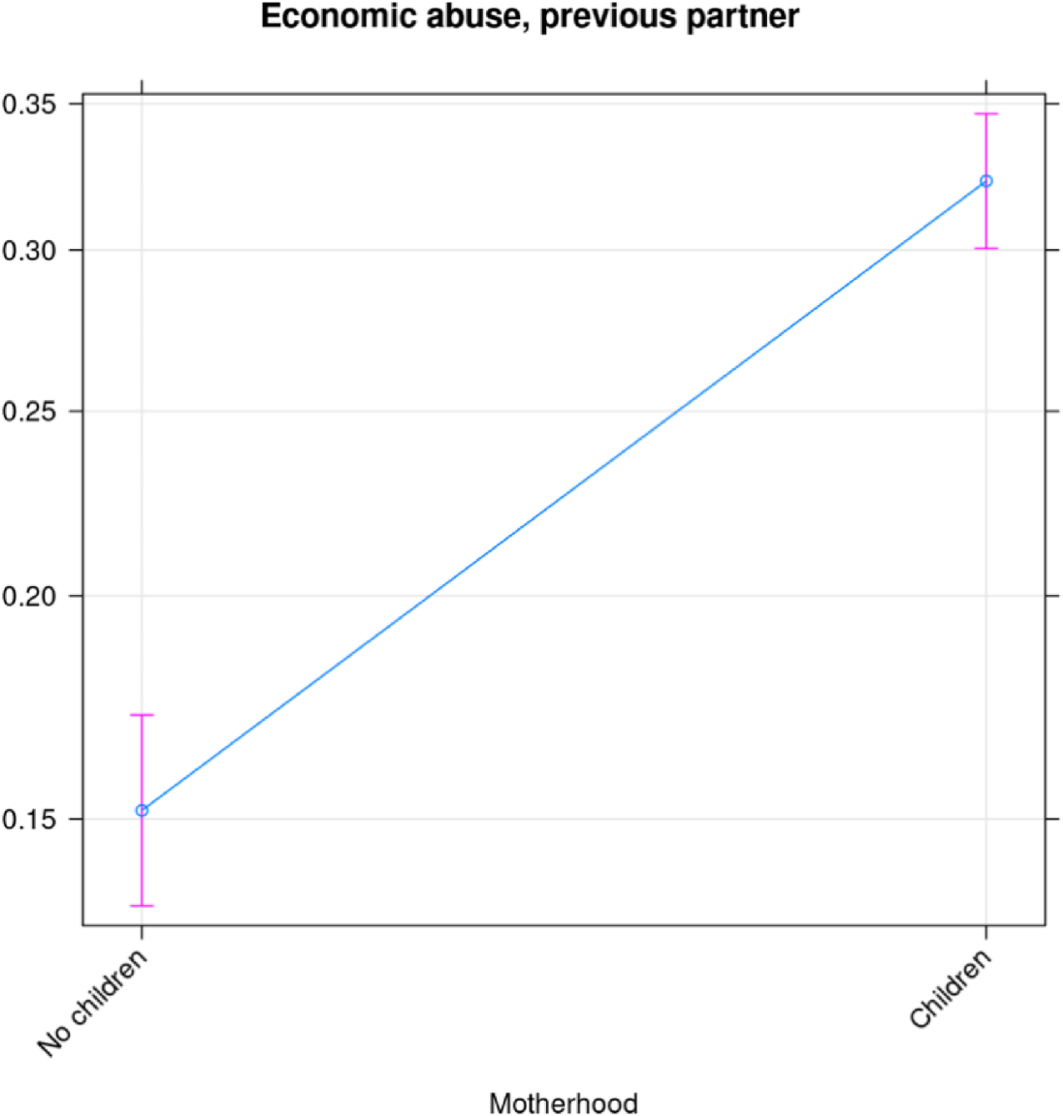
Probability of economic abuse from a former spouse/partner as a function of having children with a former partner/spouse.

We have performed a similar analysis of the risk of being exposed to economic abuse by a previous partner (Figure 5). Note, however, that the wording of the questions suggests that the economic abuse took place before separation from the previous spouse/partner. To take account of this, the categories “no children” and “only current partner” were merged into “not mother” (which most likely was the motherhood state of the woman at the time of the abuse from the previous partner) and “only previous partner” and “former and current partner” were merged into “mother.” In Figure 5, the father might be another expartner/spouse than the one who used economic abuse, in which case motherhood could occur after the abuse. However, given the very strong effect of motherhood on the probability of the outcome that seems uncommon. Figure 5 suggests that the risk of economic abuse doubles, from 15% to above 30% when the woman becomes a mother. This difference is statistically significant, as can be inferred from the nonoverlapping confidence intervals. While Figure 5 shows the probability of being exposed to economic abuse by the current partner, we have also decomposed economic abuse into exploitation, economic control, and employment sabotage, and show how each of these varies with different statuses of motherhood. Exploitation appears to be driven by having children in common, while neither the risk of economic control nor the risk of employment sabotage is significantly correlated with being a mother.

**Figure 6.**
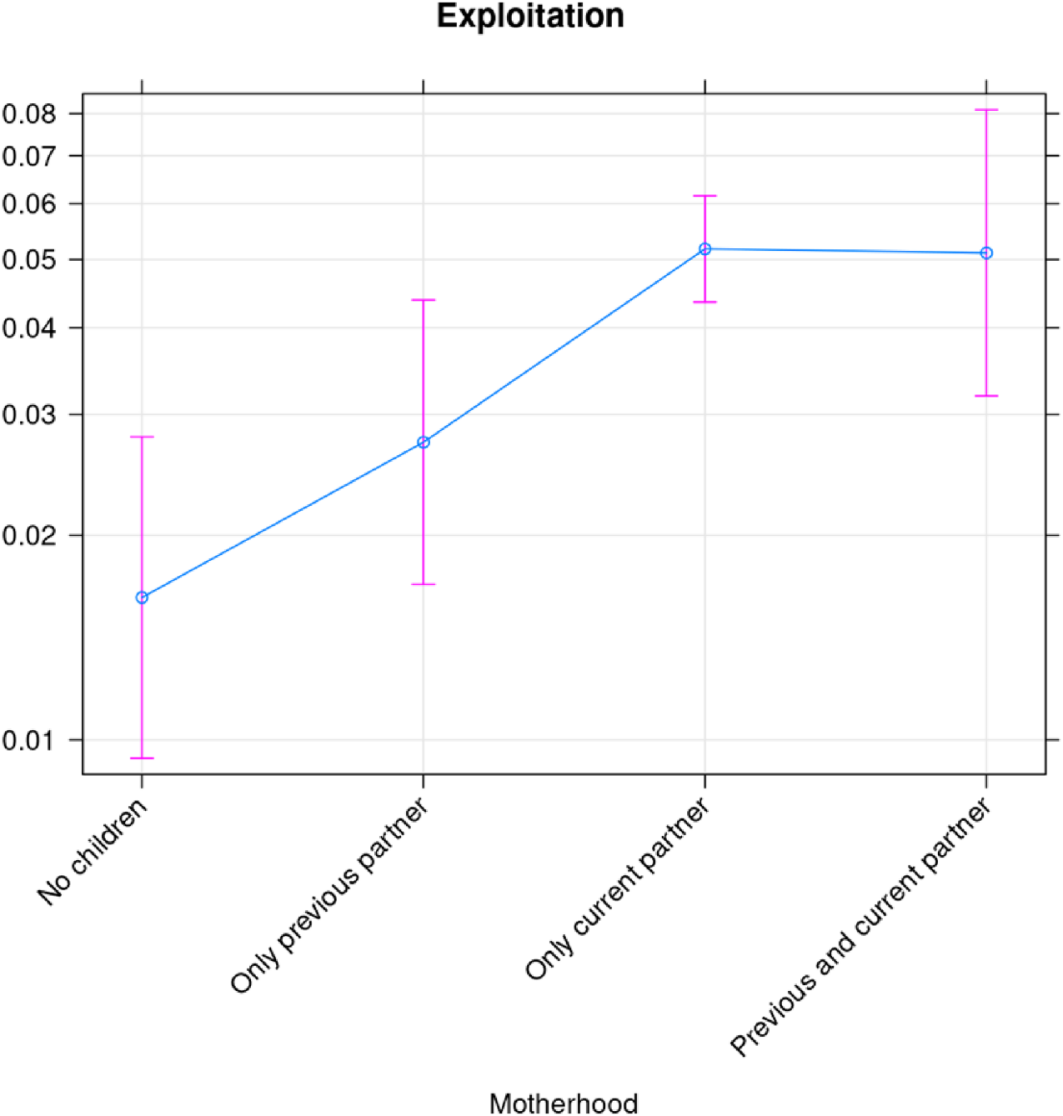
Exploitation by the current partner as a function of motherhood.

**Figure 7.**
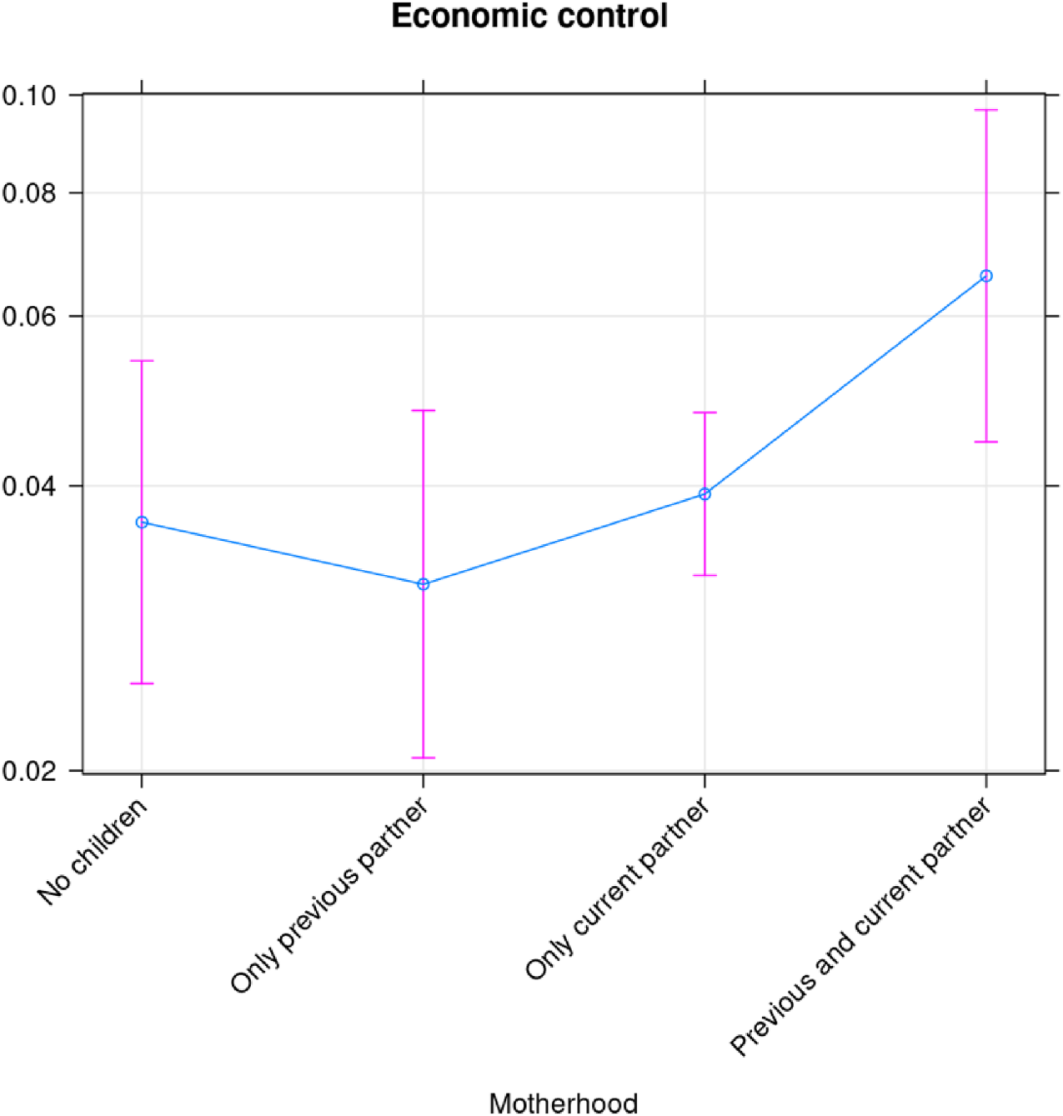
Economic control by current partner/spouse.

**Figure 8.**
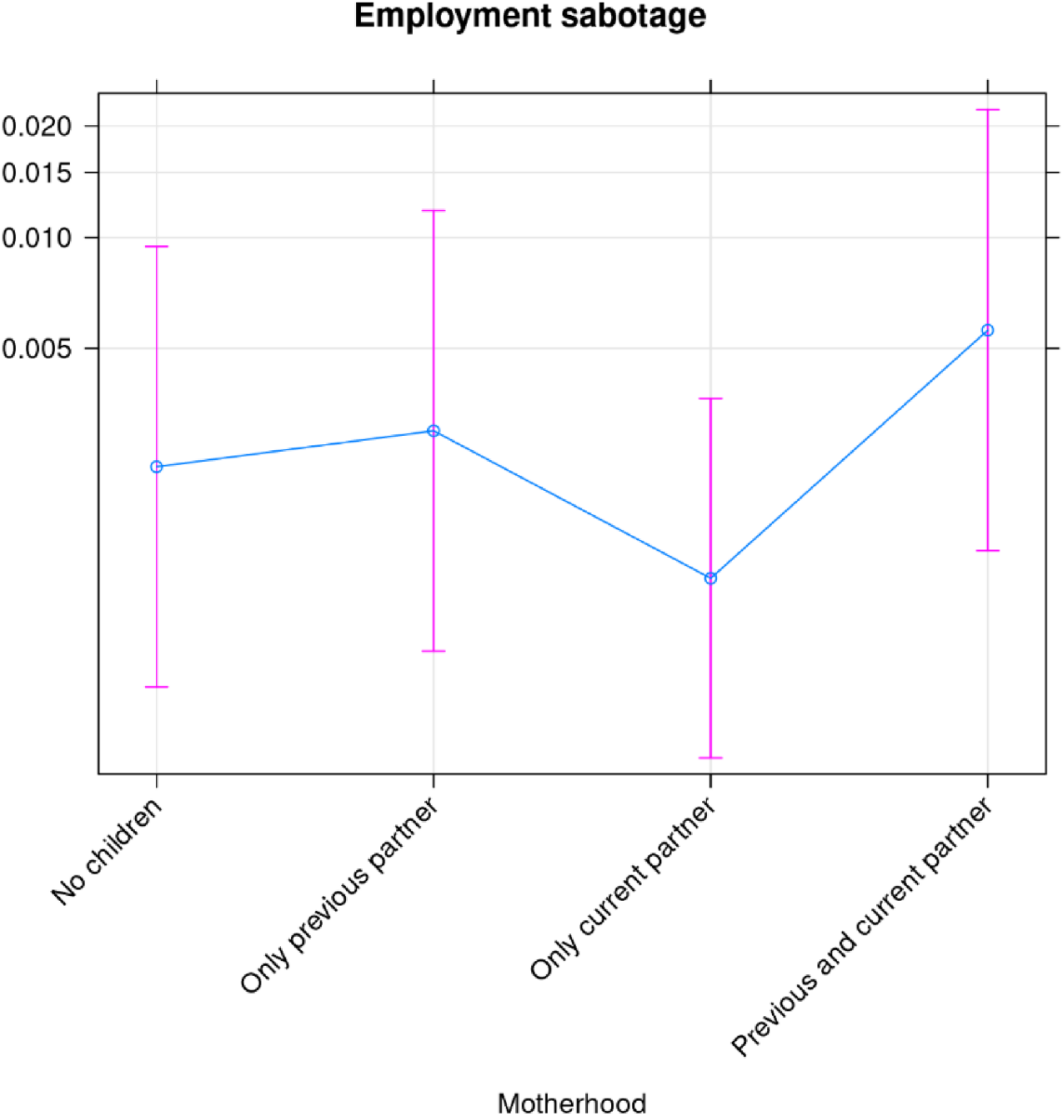
Employment sabotage by current partner/spouse.

Similar analyses for exploitation (Figure 6), economic control (Figure 7), and employment sabotage (Figure 8) from former spouses/partners, show that the risk of the former two are statistically different (higher) for mothers than for women without children, while employment sabotage is unrelated to motherhood (Figure 8).

As for the effect of socioeconomic factors, there is not much to report. The two oldest age groups (65–74 and 75–86), which both included women who have the right to age pension, are less exposed than the younger women; but education and income are not significantly correlated with economic violence.

## Discussion

The present article confirms previous research which suggests a considerable overlap between economic abuse and several other types of IPV (Gottlieb & Mahabir, 2021; Haeseler, 2013; Stylianou, 2018). An overwhelming majority (88.5%) of respondents who have experienced economic abuse have experienced more than one type. From a current partner, the correlation is strongest between economic abuse and threats (0.42). From an expartner, the correlation is stronger between economic abuse and physical violence (0.59). Since previous research suggests that 76% to 99% of survivors of IPV among service-seeking samples report having experienced economic abuse (Adams et al., 2015; Postmus et al., 2012, 2016; Stylianou et al., 2013), overlaps between different types of IPV was an expected finding. Indeed, it supports the call for increased awareness and knowledge of economic abuse among relevant authorities and IPV services (Fernqvist et al., 2023).

Low-income women without higher education report experiences of economic abuse to a slightly greater extent than women in more stable socioeconomic situations. Significant differences, however, are found between women with and without children, also when controlled for age. When a woman becomes a mother, the risk of being subjected to this type of IPV is doubled. Motherhood, thus, stands out as the single greatest risk factor for economic abuse.

The aforementioned ground-breaking Swedish study, *Captured Queen* (Lundgren et al. 2001), includes three of the five items measuring economic abuse included in our study. This enables comparison over time. Yet, the forms of abuse measured by these three items were then categorized as a form of controlling behavior/psychological violence, in line with established perspectives in violence research at the time *Captured Queen* was conducted. Compared to our present study, the results of these three questions are strikingly similar: 11% in the previous study reported having been denied influence over the economy by a former partner, compared to 12% in the present study reported being prevented from using money or to purchase things. Ten percent in the previous study reported that a former partner purposedly destroyed their belongings, as compared to 8% in the present study. Employment sabotage from a former partner was reported by 3% in the previous study and 5% in the present. In addition to economic control and employment sabotage, assessed in *Captured Queen* (Lundgren et al., 2001) the present study includes two items measuring exploitation, the third subcategory of economic abuse included in the instruments SEA-28 (Adams et al., 2008) and SEA-12 (Postmus et al., 2016). The difference in prevalence between the three subcategories of economic abuse is an unexpected finding. While economic control is the most researched form of economic abuse internationally (Postmus et al., 2020), economic exploitation stands out as the most prevalent form in this Swedish sample. Nearly one in five (17%) reports that a former partner/spouse used their money to trade for himself. Six percent report having been forced by a former partner/spouse to borrow money from others. As it appears from our survey, few Swedish men force their women partners not to work outside the home or are financially controlling. In the Philippines, by contrast, employment sabotage is suggested to be the predominant form of economic abuse (Antai et al., 2014). Significantly more often, Swedish men instead appear to use exploitative tactics. A possible interpretation of the comparatively high level of economic exploitation of women in Sweden is that it should be understood in relation to the established Nordic norm of the dual-income family and the large proportion of women participating in the workforce (Swedish Gender Equality Agency, 2023). Still, this important finding warrants further inquiry and analysis.

As with other types of violence included in the survey, women report all forms of economic violence to a significantly higher degree from former partners, in comparison with current partners. One in four (25%) respondents report having been subjected to at least one of the examples of economic abuse included in the survey, from a former partner/spouse, in comparison to one in 12 (8%) from a current partner/spouse. Do these results reflect an increase in violence(s) or an increased capacity to recognize and disclose postseparation? In previous research, there is support for both interpretations. If economic abuse does increase postseparation—which some previous research also suggests—more research is needed on the mechanisms and policies relevant in the separation context. As mentioned in the literature review section, some types of economic abuse obviously arise following separation, in the context of shared parental responsibility (c.f. Fernqvist & Sépulchre, 2022). From our material we cannot determine whether the economic abuse by former partners reported refers to past experiences of abuse while still in the relationship (and in the shared household) or actually postseparation. Moreover, we do not know if the violence has been exerted by one expartner only, or if it occurred in several relationships. In-depth interviews with survivors would contribute to determining more about the contextual and temporal factors associated with economic IPV.

### Recommendations

There are still several unresolved issues that warrant further inquiry, and we recommend future research utilize mixed methods. Qualitative research on economic abuse remains of great importance, not least because such studies can capture more expressions of violence with more nuances than can be revealed in a survey. Qualitative data gathering and analysis can explore how this type of IPV is experienced, in which context it was perpetuated, and which strategies survivors use to handle it. In addition, informants tend to disclose more forms of economic abuse in qualitative interviews than they report within questionnaires, which illuminates the importance of using multiple methods (Sharp-Jeffs, 2015). Since only five items measuring economic abuse were included in this study and economic abuse can be exerted in numerous ways, there is also a need for more quantitative research. SEA-12 should thus be tested in Sweden (and other countries). Furthermore, we concur with Kaittila et al. (2024), who stress that existing measurements to assess economic abuse focus on the extent of this type of IPV within relationships. For the purpose of assessing economic abuse postseparation, SEA-12 may be insufficient and in need of further refinement.

Another crucial issue is how different agencies frame and respond to the problem—implications for policy and practice. As a suggestion, SEA-12 could be supplemented with additional questions regarding the institutional and intersectional conditions and effects of economic abuse. There is a need to critically examine the welfare state's handling of economic abuse on a system level. Policies and professional discourses and practices within family law, social services, and social insurance may aggravate inequalities and increase abusers’ scope for action, several studies suggest (c.f. Tegler et al., 2023). Obviously, contexts and implications of economic violence vary between different groups of survivors. Accordingly, further research should take into consideration and analyze how the intersections of survivors’ social positions, disadvantages and radically different life situations, and resources impact their possibilities of having their experiences validated and the support they need to be provided (Anitha, 2019; Bullock et al., 2020; Crenshaw, 1989; Walby et al., 2014).

Ultimately, there is also a need for theoretical development, conceptual clarity, and further refinement as regards what to include, and how to use the term economic abuse and similar concepts. For example, coerced debt is included in SEA-12 as a form of economic exploitation but is in some research defined as a form of coercive control and examined in its own right (Adams et al., 2020; Littwin, 2012). Also, *material abuse/violence* (to intentionally damage the victim's belongings), is sometimes used interchangeably with financial/economic abuse, such as in the definition “Material abuse means the misuse of a person's property or financial resources” (Law Insider, 2023). The item is not included in SEA-12, but in *Captured Queen* (Lundgren et al., 2001) as a form of psychological violence and in our present study as a form of economic abuse.

## Conclusion

The present article is the first which is based on a prevalence study measuring economic abuse as a distinct type of men's violence against women in the Nordic context. It aims to analyze the prevalence and characteristics of men's intimate partner economic abuse against women in Sweden and reveals partly unexpected findings, which have implications for scholarship, policy, and practice. A major conclusion is that becoming a mother doubles the risk of experiencing this type of IPV. In contrast, there is no significant correlation between economic abuse and education or income. The overall prevalence of economic abuse is 13%, with economic exploitation being the most common form of economic abuse. However, as many as one in four women report having been subjected to economic abuse by a former partner. Rather than occur in isolation, different types of IPVs tend to cooccur. Notable is the overlap in our study between, specifically, economic and physical abuse.

It is crucial to recognize and analyze all types of IPVs in order to address them with greater efficiency. When this type of IPV is overlooked in research—which until recently has been the case in Sweden—it hinders a more developed analysis of men's violence against women. Moreover, when economic abuse is overlooked by authorities and legislators, it may contribute to a deepening of intersecting inequalities in society and to the persistence of men's violence against women as a major issue.
